# The Usefulness of Diagnostic Panels Based on Circulating Adipocytokines/Regulatory Peptides, Renal Function Tests, Insulin Resistance Indicators and Lipid-Carbohydrate Metabolism Parameters in Diagnosis and Prognosis of Type 2 Diabetes Mellitus with Obesity

**DOI:** 10.3390/biom10091304

**Published:** 2020-09-09

**Authors:** Katarzyna Komosinska-Vassev, Olga Gala, Krystyna Olczyk, Agnieszka Jura-Półtorak, Paweł Olczyk

**Affiliations:** 1 Department of Clinical Chemistry and Laboratory Diagnostics, Faculty of Pharmaceutical Sciences in Sosnowiec, Medical University of Silesia in Katowice, 41-200 Sosnowiec, Poland; ogala@wp.pl (O.G.); olczyk@sum.edu.pl (K.O.); ajura@sum.edu.pl (A.J.-P.); 2 Department of Community Pharmacy, Faculty of Pharmaceutical Sciences in Sosnowiec, Medical University of Silesia in Katowice, 41-200 Sosnowiec, Poland; polczyk@sum.edu.pl

**Keywords:** adipocytokines, obesity, type 2 diabetes mellitus, adropin, irisin, vaspin, metformin therapy

## Abstract

The quantitative analysis of selected regulatory molecules, i.e., adropin, irisin, and vaspin in the plasma of obese patients with newly diagnosed, untreated type 2 diabetes mellitus, and in the same patients after six months of using metformin, in relation to adropinemia, irisinemia and vaspinemia in obese individuals, was performed. The relationship between plasma concentration of the adipocytokines/regulatory peptides and parameters of renal function (albumin/creatinine ratio—ACR, estimated glomerular filtration rate—eGFR), values of insulin resistance indicators (Homeostatic Model Assessment of Insulin Resistance (HOMA-IR2), Homeostatic Model Assessment of Insulin Sensitivity (HOMA-S), Homeostatic Model Assessment of β-cell function (HOMA-B), quantitative insulin sensitivity check index (QUICKI), insulin), and parameters of carbohydrate-lipid metabolism (fasting plasma glucose—FPG, glycated hemoglobin—HbA_1C_, estimated glucose disposal rate—eGDR, fasting lipid profile, TG/HDL ratio) in obese type 2 diabetic patients was also investigated. Circulating irisin and vaspin were found significantly different in subjects with metabolically healthy obesity and in type 2 diabetic patients. Significant increases in blood levels of both analyzed adipokines/regulatory peptides were observed in diabetic patients after six months of metformin treatment, as compared to pre-treatment levels. The change in plasma vaspin level in response to metformin therapy was parallel with the improving of insulin resistance/sensitivity parameters. An attempt was made to identify a set of biochemical tests that would vary greatly in obese non-diabetic subjects and obese patients with type 2 diabetes, as well as a set of parameters that are changing in patients with type 2 diabetes under the influence of six months metformin therapy, and thus differentiating patients′ metabolic state before and after treatment. For these data analyses, both statistical measures of strength of the relationships of individual parameters, as well as multidimensional methods, including discriminant analysis and multifactorial analysis derived from machine learning methods, were used. Adropin, irisin, and vaspin were found as promising regulatory molecules, which may turn out to be useful indicators in the early detection of T2DM and differentiating the obesity phenotype with normal metabolic profile from T2DM obese patients. Multifactorial discriminant analysis revealed that irisin and vaspin plasma levels contribute clinically relevant information concerning the effectiveness of metformin treatment in T2D patients. Among the sets of variables differentiating with the highest accuracy the metabolic state of patients before and after six-month metformin treatment, were: (1) vaspin, HbA1c, HDL, LDL, TG, insulin, and HOMA-B (ACC = 88 [%]); (2) vaspin, irisin, QUICKI, and eGDR (ACC = 86 [%]); as well as, (3) vaspin, irisin, LDL, HOMA-S, ACR, and eGFR (ACC = 86 [%]).

## 1. Introduction

Type 2 diabetes (T2DM) is a metabolic disease that is directly caused by an impairment of insulin secretion and/or abnormalities in insulin action. The strongest factors that particularly predispose to the increase of the incidence of metabolic diseases, including T2DM, are overweight and obesity. White adipose tissue that is the source of multiple metabolically important proteins known as adipocytokines, has been recognized as an active participant in energy metabolism and body′s homeostasis [1]. These substances are considered to be the link between the adipose tissue and metabolic disorders and they are most likely involved in the development of insulin resistance and the etiopathogenesis of type 2 diabetes. In obesity, accumulated fat tissue, depending on the current metabolic status, is characterized by a different secretory profile [2,3]. Among the recently identified regulatory peptides secreted by both adipose tissue and peripheral organs (mainly liver and skeletal muscle), which play an important role in the pathogenesis of metabolic changes in the course of obesity associated with type 2 diabetes, adropin, irisin, and vaspin play an important role [4,5,6,7,8]. The first of them, **adropin**, is a secreted peptide translated from the *Energy Homeostasis Associated* (*ENHO*) gene linked to metabolic control. This is a relatively newly identified regulatory protein critically involved in the regulation of carbohydrate metabolism by affecting glucose-dependent insulin release. However, it is still not clear whether adropin is a peptide hormone regulated by signals of metabolic state, including fasting and feeding. Adropin deficiency has been connected with obesity, insulin resistance, and type 2 diabetes; nevertheless, the significance of this fact with respect to metabolic diseases that are associated with obesity, remains unclear [9,10,11,12]. **Irisin**, called an anti-diabetic hormone, regulates fat metabolism by converting white adipocytes into brown ones [13]. That protein has been found being locally produced in peripheral tissues, where it may act as a regulator of energy metabolism [14,15]. Irisin inhibits fat accumulation by increasing energy expenditure and the expression of specific enzymes. Thus, it plays a suppressor role in preadipocyte differentiation and mass increases of adipose tissue. Instead, it stimulates the expression of genes that are associated with myocyte differentiation and muscle tissue growth [16]. The third of the assessed adipokines, **vaspin**, is a protein that is produced by subcutaneous and visceral adipose tissue [17]. Previous research indicates that the expression of the gene encoding vaspin and its plasma concentration may be associated with insulin resistance in the course of obesity and type 2 diabetes. Therefore, vaspin is probably involved in glucose metabolism significantly improving glucose tolerance [18,19]. According to recent studies, there is a positive correlation between plasma vaspin levels and parameters that are associated with metabolic syndrome and type 2 diabetes [20,21]. The hormone may be a predictor of metabolic syndrome, independent of obesity [22]. There is a strong likelihood that low concentration of this adipokine is associated with insufficient glycemic control in patients due to its effect on insulin sensitivity and glucose metabolism. Therefore, vaspin probably plays a compensatory role in insulin resistance accompanying the diabetes [23,24,25]. Flehming et al. included vaspin into a group of adipokines showing association with insulin sensitivity and lipid metabolism [22].

Current research indicates that adipocytokines are involved in the pathogenesis of metabolic disorders that are associated with obesity and may be used as markers to assess the therapy results. Metformin is the first-line medication for the treatment of type 2 diabetes, particularly in overweight and obese people. Li et al. found that this type of biguanide increase intramuscular FNDC5 expression and promote irisin release from murine skeletal muscle into the circulation [26]. Peroxisome proliferator-activated receptor γ co-activator 1α (PGC-1α) was found to be a critical regulator in this process [27]. Metformin was also found as a confounding factor with regards to the decrease of circulating vaspin levels, possibly through its suppressive effect on hepatic glucose production in overweight women, leading to an improvement in insulin sensitivity [28]. However, different findings were reported in animals. A study by Gonzales et al. showed that metformin increased vaspin expression in adipose tissues in rats [29]. The effect of metformin on vaspin might be different between humans and rodents and more research is needed in order to understand the mechanism by which metformin affects the plasma profile of this adipokine.

Moreover, metformin treatment was found to inhibit adenylate cyclase resulting in reduction of cAMP level and phosphorylation of PKA substrates including IP3R, leading to the suppression of hepatic glucagon signaling [30]. The current in vitro studies demonstrate that adropin suppresses glucose production in primary hepatocytes, sharing molecular mechanisms underlying metformin’s actions on reducing hepatic glucose production. In addition to sensitizing insulin intracellular signaling, adropin may antagonize glucagon signaling pathway in reducing hyperglycemia [31]. These signaling actions appear to underlie peptide adropin’s therapeutic potential of suppressing fasting hyperglycemia and improving glycemic control in obesity-associated type-2 diabetes [31]. As with adropin, the therapeutic use of irisin and vaspin are also being considered to be adjunctive therapy for obesity and diabetes.

Taking into account the beneficial effects of all analyzed molecules—adropin, irisin, and vaspin—in regulating carbohydrate-lipid metabolism in obesity, and the fact that all of them were considered as a new therapeutic option in patients with obesity and type 2 diabetes mellitus [19,32,33,34], the aim of this study was to quantify adropin, irisin, and vaspin in the blood plasma of obese patients, with newly diagnosed type 2 diabetes before undertaking pharmacological treatment and in the same patients after six-month metformin therapy, in relation to adropinemia, irisinemia, and vaspinemia of obese subjects without diabetes. Correlations between adropin, irisin, and vaspin and (1) the parameters of renal function (albumin/creatinine ratio—ACR, estimated glomerular filtration rate—eGFR), (2) values of insulin resistance indicators (Homeostatic Model Assessment of Insulin Resistance (HOMA-IR2), Homeostatic Model Assessment of Insulin Sensitivity (HOMA-S), Homeostatic Model Assessment of β-cell function (HOMA-B), quantitative insulin sensitivity check index (QUICKI), insulin), and (3) parameters of carbohydrate-lipid metabolism (fasting plasma glucose—FPG, glycated hemoglobin—HbA_1C_, estimated glucose disposal rate—eGDR, fasting lipid profile, TG/HDL ratio) were also evaluated. Based on the discriminant analysis, the assessment and visualisation of correlations between adropin, irisin, and vaspin and selected biochemical parameters in a three-variable system were performed.

An attempt was also made to create biochemical diagnostic panels, which, based on irisinemia and/or vaspinemia, and selected indicators of the patient′s metabolic status (HbA1c, HDL, LDL, TG, ACR, eGFR, glucose, insulin, QUICKl, HOMA-IR, HOMA-S, HOMA-B, and eGDR), undergo the greatest changes during the applied pharmacological therapy. The diagnostic panels, which greatly differentiate the patient′s metabolic condition before and after treatment, could confirm the effectiveness of the therapy used or indicate the need for its modification.

An innovative aspect of the work is the combination of diagnostic methods and modern methods based on the use of IT tools, such as discriminant analysis and multi-parameter biomedical data analysis, derived from machine learning methods.

## 2. Experimental Section

Venous blood and urine samples were obtained from 40 obese patients of both sexes aged from 47 to 72 years, with newly diagnosed type 2 diabetes (recognized within three month) and subjected to behavioral treatment. T2DM diagnosis was based on the Standards of Clinical Care in Diabetes by the Polish Diabetes Association, as follows: fasting plasma glucose (FPG) ≥ 126 mg/dL obtained on at least two occasions, or 2 h blood glucose ≥ 200 mg/dL by oral glucose tolerance test. All individuals included into the study had no microvascular complications. Retinopathy was excluded by ophthalmoscopic examination and retinal fluoresceinangiography. Nephropathy was monitored by the determination of urinary albuminuria, urinary albumin/creatinine ratio (ACR), and eGFR. Diabetic nephropathy was defined as the presence of microalbuminuria and overt albuminuria. Urinary albumin excretion was investigated in order to assess diabetic nephropathy. Urinary albumin excretion of 30–300 μg/mg was regarded as microalbuminuria, and urinary albumin excretion of more than 300 μg/mg was regarded as overt albuminuria. Patients were also questioned about sensory, motor, and autonomic symptoms. A clinical history regarding any other concurrent etiology for neuropathy was evaluated. A standard neurological examination included an evaluation of knee and ankle reflex activity and feet sensation with monofilament and vibration. The exclusion criteria were also: patients with other types of diabetes; patients with gestational diabetes; subjects with autoimmune diseases; patients treated with glucocorticosteroids, adrenocorticotropic hormone or interferons; subjects with a prior stroke or myocardial infarction; patients with unstable angina; patients with class III and class IV heart failures according to New York Heart Association (NYHA); patients with liver failure; and, subjects with kidney failure as well as patients with hyperthyroidism and other endocrine and metabolic diseases. Patients qualified for the study underwent anthropometric measurements, including, among others, body mass index (BMI—calculated as weight (in kg)/height (in m^2^) as well as waist to hip ratio (WHR) assessment. Basic hematological and biochemical blood tests (renal and liver function analysis, fasting glucose, glycated hemoglobin content, insulin level, and fasting lipid profile) were performed for every person qualified for the examination. In addition, urine creatinine and albumin levels were measured in first-morning urine samples. Albumin/creatinine ratio (ACR) was calculated by dividing albumin concentration in micrograms by creatinine concentration in milligrams in order to assess the severity of urinary albumin excretion. The value of the estimated glomerular filtration rate (eGFR) was calculated on the basis of a simplified four-Variable MDRD equation: GFR in mL/min. per 1.73 m^2^ = 175 × serumCr^−1.154^ × age^−0.203^ × 1.212 (if patient is black) × 0.742 (if female). Insulin resistance was evaluated using indirect indicators of tissue sensitivity to insulin: Homeostatic Model Assessment of Insulin Resistance (HOMA-IR2), Homeostatic Model Assessment of Insulin Sensitivity (HOMA-S), and Homeostatic Model Assessment of β-cell function (HOMA-B) index, calculated based on the formula available at: www.dtu.ox.ac.uk/homacalculator/. HOMA-IR = fasting insulin (μU/mL) × fasting glucose (mg/dL)/405). The QUICKI insulin resistance index was calculated with the formula QUICKI = 1/(log [fasting insulin (mU/l)] + log [fasting plasma glucose (mg/dL)]. As an indirect exponent of insulin resistance estimating glucose uptake by target tissues, the estimated glucose disposal rate (eGDR) was calculated on the basis of the formula, taking into account the degree of glycemic control, anthropometric data describing abdominal obesity and the presence or absence of hypertension:

$$eGDR\;\left(\frac{\frac{mg}{kg}}{min}\right)=24.31-12.22\;\left(WHR\right)-3.29\;(hypertension\;0/1)-0.57\;\left(HbA1c\right)$$

eGDR correlates with the content of abdominal fat and the assessment of insulin resistance using hyperinsulin euglycemic clamp. An eGDR value < 7.5 (mg/kg/min), was considered to be an indicator of reduced tissue sensitivity to insulin. The TG/HDL ratio was analyzed as an indirect surrogate marker of insulin resistance. Anthropometric parameters, indicators of renal function, insulin sensitivity parameters, and biochemical laboratory tests in patients with type 2 diabetes were assessed twice: before starting treatment and six months after the metformin therapy. Patients received metformin in monotherapy. The dose of oral metformin administration was 1500 mg or 2550 mg three times a day. Moreover, all of the diabetic patients received advice and consultation for lifestyle modifications in diet and physical activity.

The reference material for the study were blood samples obtained from 20 obese subjects without diabetes, both sexes, at the age corresponding to the age of patients with diabetes. Subjects in the control group were screened by means of medical history, physical examination, and laboratory analyses. We included subjects who did not undergo surgery or hospitalization during the year prior to the study, and were not pharmacologically treated during the study. The exclusion criteria were also the clinical evidence of inflammatory disease, the presence of diabetes mellitus, cancer, or kidney and liver disease. Obese individuals without diabetes were characterized by an obesity phenotype with the so-called “normal metabolic profile”. They had normal lipid profile or only slight lipid metabolism disorders, normal blood pressure, fasting glucose < 100 mg/dL, and did not meet the criteria for diagnosing metabolic syndrome. Such a control group allowed for distinguishing the adipose tissue profile in terms of the expression of analysed adipokines, between obesity phenotype with normal metabolic profile and obesity representing phenotype with metabolic disorders closely related with the presence of T2DM. Table 1 shows the clinical characteristics of the study population including control subjects and diabetic patients.

Informed consent was obtained from all of the participants according to the ethical guidelines of the Declaration of Helsinki. The Bioethical Committee of the Medical University of Silesia in Katowice approved the research protocol that was used in this study (KNW/0022/KB1/147/10).

The plasma concentration of adropin in control and obese individuals with type 2 diabetes was measured by enzyme-linked immunosorbent kit by Phoenix Pharmaceuticals (USA). The sensitivity of the test is 0.3 (ng/mL). The intra-assay variability was less than 7 [%]. The quantitative measurement of irisin was determined with a commercially available enzyme-linked immunosorbent assay (ELISA) kit supplied by BioVendor R&D (Brno, Czech Republic), following the manufacturer’s recommendations. The test sensitivity is 1 (ng/mL) and the detection range was 0.001−5 (µg/mL). The intra-assay variability was less than 8 [%]. To assess the plasma concentration of vaspin in control subjects and patients with type 2 diabetes, an immunoenzymatic (ELISA) test that was obtained from BioVendor R&D (Brno, Czech Republic) was used. The sensitivity of the test is 0.01 (ng/mL). The intra-assay variability was less than 6 [%].

Data analyses were performed using the StatSoft, Inc. (2014), STATISTICA (data analysis software system), version 12 (https://www.statsoft.pl). The normality of distribution was verified by Shapiro-Wilk test and homogeneity of variance by Levene’s test. The data were expressed as means ± standard deviation or median and interquartile range. The Student’s *t*-test or nonparametric Mann-Whitney U-test for independent variables was used to compare the differences between diabetic patients and controls. Statistical differences between the untreated T2D patients and after a six-month of pharmacotherapy were verified by the Student′s *t*-test or nonparametric Wilcoxona test for dependent variables. The limit of statistical significance was set at *p* < 0.05. The relationship between adropin, irisin, and vaspin and insulin resistance indices, renal function parameters, and carbohydrate-lipid metabolism parameters was also assessed. The strength of association between two variables was assessed by Spearman′s rank correlation coefficient. Additional correlation analyses between % change of adropin, irisin, and vaspin with % change of clinical and metabolic parameters were also performed.

The key element of the study was discriminant analysis using bioinformatics tools, as it allowed to decide which biochemical or anthropometric variables best discriminate between data from patients with T2D as compared to healthy people, and also which of the parameters mentioned clearly differ in patients before and after treatment. The three-parameter assessment and visualization of the relationship between the examined adipocytokines and selected indicators of renal function, parameters of tissue insulin resistance, and indicators of lipid-carbohydrate metabolism were carried out based on discriminatory analysis. The mahalanobis type of analysis was chosen, which allows for obtaining the best group separability. This analysis was associated with the division of all quantitative results from healthy individuals (one group) and results obtained in the group of patients, both before and after 6 months of therapy (second group). These two groups were then divided into test set (rounded to the total value 1/3 of the total data) and learning dataset (rounded to the total value 2/3 of the total data). The accuracy (ACC) values were assessed defined as ACC = (TP + TN)/(FN + FP + TN + TP), where TP represents true positive, TN represents true negative, FN represents false negative, and FP represents false positive. ACC values exceeding 50 [%] quantitatively confirm the separability of two data groups. Thus, they confirm the possible separation of obese non-diabetic patients from obese T2D patients, based on the three variables studied and, analogously, the distinction between T2D patients before and after treatment.

Multi-parameter analysis of the relationship between irisin and/or vaspin and the selected combination of 13 biochemical indicators was another aspect of research using artificial intelligence. The IT analysis of the data was carried out using the Matlab package with Signal Processing Toolbox (Matlab: Version 7.11.0.584, R2010b, Java VM Version: Java 1.6.0_17-b04 with Sun Microsystems Inc.; Signal Processing Toolbox: Version 7.1) on a PC running Windows 7 Professional, 64-bit with the Intel Core i7-4960X CPU @ 3.60 GHz.

## 3. Results

Comparative analysis of biochemical parameters revealed that there were no significant differences as regards TG, HDL, insulin, HOMA-S, QUICKI, as well as urinary albumin and creatinine levels between control subjects and type 2 diabetic patients. After the six-month of metformin treatment, significant improvement in diastolic blood pressure and lipid profile (total cholesterol and LDL cholesterol) was parallel to the improvement in insulin sensitivity expressed by the decrease in HOMA-IR2 levels. No distinct improvement was observed in glucose and HbA1c level after the six-month metformin therapy. As a result of the research, no significant differences in the plasma **adropin** levels were observed in T2DM individuals after six-month metformin treatment compared to the situation before treatment, as well as to the plasma adropin values in obese control subjects. Table 2 provides a report on plasma adipocytokines in control subjects and T2DM patients at baseline and after six-month of pharmacological treatment with metformin.

We have found no significant effect of gender on the adropin, irisin, and vaspin plasma levels in obese T2DM patients. On the other hand, in the group of obese control subjects, men had statistically significantly higher plasma levels of adropin than women.

We next examined the correlations between plasma adipocytokines and renal function indices, insulin resistance indicators, and carbohydrate-lipid metabolism parameters in diabetic patients, which were summarized in Table 3.

The plasma adropin displayed positive correlations with TG concentration (*r* = 0.479, *p* = 0.003) and TG/HDL ratio (*r* = 0.383, *p* = 0.021) in the T2DM patients before metformin therapy.

The conducted studies showed that, in the blood serum of obese T2DM patients, both before and after the pharmacotherapy, **irisin** concentrations were significantly higher when compared to the values of this protein in healthy obese people. Moreover, significantly higher concentrations of the assessed regulatory molecule were demonstrated in patients after half-year metformin treatment, when compared to the situation before the pharmacotherapy inception. In the pre-treatment group, statistically significant negative correlations were found between plasma irisin and eGFR (*r* = −0.304, *p* = 0.045) and HbA1c (*r* = −0.430, *p* = 0.008). In the group after half-year metformin therapy, a positive correlation between plasma irisin and QUICKI (*r* = 0.349, *p* = 0.039) was stated.

In relation to the next assessed regulatory molecule, significantly lower plasma **vaspin** level was found in obese newly diagnosed T2DM patients before the therapy implementation, as compared to healthy controls. Six-month metformin treatment resulted in a 48 [%] increase in plasma vaspin level compared to the situation before pharmacological intervention. In pre-treatment obese T2DM patients, plasma vaspin displayed four statistically significant correlations: positive with HOMA-IR (*r* = 0.437, *p* = 0.020), insulin concentration (*r* = 0.491, *p* = 0.007), and HOMA-B (*r* = 0.352, *p* = 0.006), and a negative with HOMA-S (*r* = −0.445, *p* = 0.017). In the study group, after six-month metformin treatment, a negative association was observed between plasma vaspin and QUICKI (*r* = −0.372, *p* = 0.025).

Additional correlation analysis allowed finding that percentage change in plasma vaspin concentration during metformin treatment was accompanied by a significant percentage decrease in HOMA-IR (*r* = −0.619; *p* = 0.032) and HOMA-B (*r* = −0.464; *p* = 0.015). On the other hand, a tendency has been found between the change in insulin sensitivity parameter i.e., HOMA-S (*r* = 0.796, *p* = 0.069) in response to metformin treatment and the change in plasma vaspin concentration following conventional therapy for type 2 diabetes.

The effect of six-month metformin therapy was also analyzed in our study in relation to the parameters of clinical characteristics of patients. Significant improvement in diastolic blood pressure and lipid profile (total cholesterol and LDL cholesterol) was parallel to the improvement in insulin sensitivity expressed by the decrease in HOMA-IR2 levels. However, using metformin alone during the first six months from the implementation of pharmacotherapy did not improve glycemic control in diabetic patients. Thus, the implementation of initial therapy with oral antidiabetic agent was not enough to achieve glycemic goals, however it provided to be effective to improve lipid parameters.

Based on the initial statistical analysis, a set of 10 biochemical variables that greatly differ in healthy subjects and T2DM patients: HOMA-IR, QUICKl, ACR, eGFR, HbA1c, eGDR, HOMA-B, HOMA-S, insulin concentration, and TG/HDL ratio, which, together with the plasma levels of adropin, irisin, and vaspin, was selected to further tests using three-parameter discriminant analysis. This method allowed a quantitative assessment of the relationship between individual variables in the system of three selected parameters. A summary of quality indicators of classification models in a three-parameter system regarding the selection of the most important biochemical parameters that differ significantly in obese subjects without diabetes and obese patients with T2DM is presented in Table 4 (part A). The applied discriminant analysis confirmed the statistical significance of all proposed three-parameter diagnostic panels as factors discriminating healthy subjects from obese patients with type 2 diabetes. The most important biochemical parameters in constructed diagnostic panels turned out to be plasma adropin, HOMA-B, and HOMA-S (ACC = 95 [%]), plasma adropin, irisin, and vaspin (ACC = 94 [%]), as well as plasma irisin, index QUICKI, and HOMA-IR (ACC = 90 [%]).

**Figure 1:**
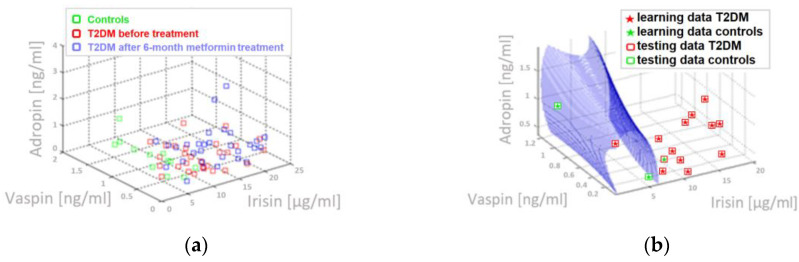

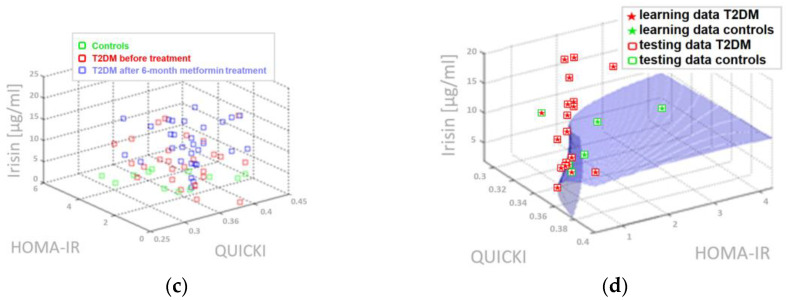
Three-parameter discriminant analysis, which allow to differentiate subjects with metabolically healthy obesity and T2DM obese patients: (**a**) Adropin, irisin and vaspin data set obtained from all examined individuals (**b**) Graph of the discriminatory curve which based on adropin, irisin and vaspin allows for separation between healthy obesity and T2DM patients with **ACC = 94 [%]** (**c**) Irisin, QIUCKI, and HOMA-IR data set obtained from all examined individuals (**d**) Graph of the discriminatory curve which based on irisin, QIUCKI and HOMA-IR allows for separation between healthy obesity and T2DM patients with **ACC = 90 [%]**.

Figure 1 presents examples of performed three-parameter discriminant analysis visualization, which allow to differentiate the patients with metabolically healthy obesity and T2DM obese patients.

In addition, the performed IT analyzes have allowed proposing diagnostic profiles based on the variables that significantly differ in patients with diabetes before and after the implementation of metformin therapy. Performed analysis has shown that the most important combination of three parameters that significantly differentiates patients before and after treatment was the diagnostic panel containing irisin, HbA1c, and eGDR (ACC = 80 [%]), and a model based on the plasma adropin, irisin, and vaspin (ACC = 69 [%]).

The quality indicators of classification models in a three-parameter system regarding the selection of the most important biochemical parameters differing in obese patients with T2DM before and after pharmacological treatment are presented in Table 4 (part B).

**Figure 2:**
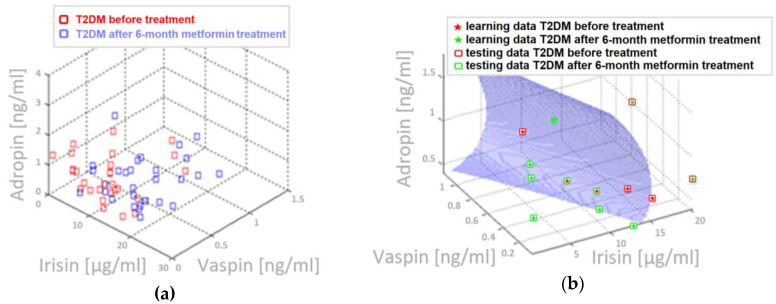

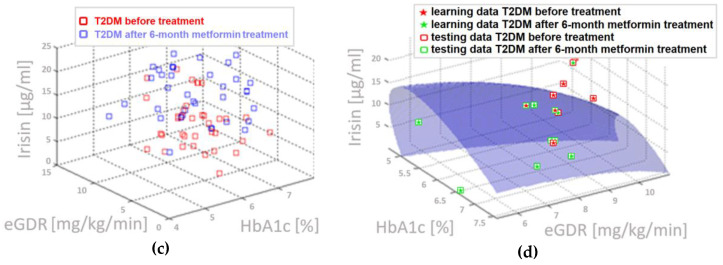
Three-parameter discriminant analysis, which allow to differentiate metabolic state of T2DM patient before treatment and after 6-month metformin therapy: (**a**) adropin, irisin, and vaspin data set obtained diabetic patients (**b**) graph of the discriminatory curve which based on adropin, irisin and vaspin allows for separation between metabolic state od diabetic patients before and after metformin treatment; **ACC = 69 [%]** (**c**) irisin, HbA1c, and eGDR data set obtained from diabetic patients; and, (**d**) graph of the discriminatory curve, which, based on irisin, HbA1c, and eGDR, allows for separation between metabolic state od diabetic patients before and after metformin treatment; **ACC = 80 [%]**.

Examples of performed three-parameter discriminant analysis visualization, which allow to differentiate the patients with metabolically healthy obesity and T2DM obese patients, are presented in Figure 2.

**Figure 3:**
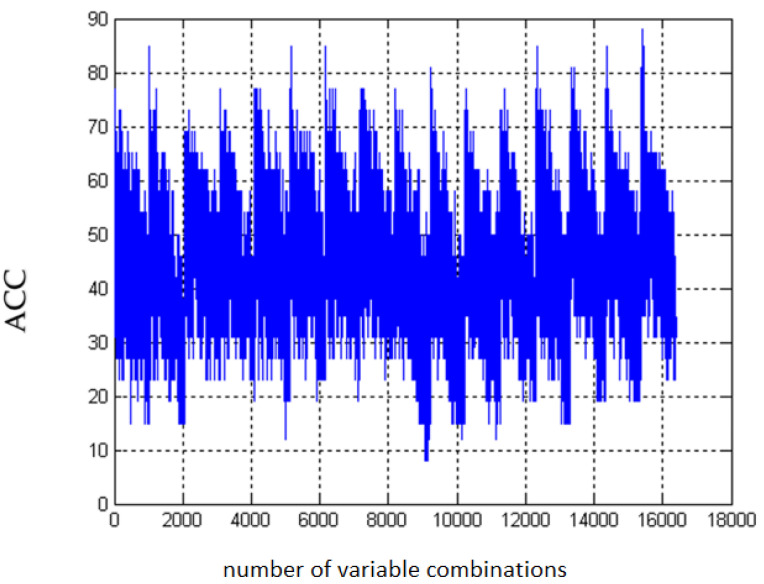
A cumulative graph of ACC value changes for all variable combinations of selected variables (HbA1c, HDL, LDL, TG, ACR, eGFR, glucose, insulin, QUICKI, HOMA-IR, HOMA-S, HOMA-B, and eGDR) with always present vaspin. The number of all analyzed combinations of selected parameters was specified as 2^14^ − 1 = 16383 classifications, among which the diagnostic panels with the highest ACC value were selected.

In search for complex relationships between the analyzed regulatory molecules—adropin, irisin, and vaspin, and selected metabolic and biochemical parameters during the treatment of type 2 diabetes, advanced methods of **multivariate analysis** were also used. Figure 3 presents an example of performed classifications.

As part of this task, the impact of several factors for which previous analyzes confirmed statistical significance as parameters significantly changing after the implementation of therapeutic treatment in obese T2DM patients, was assessed, including the level of irisin and vaspin, HbA1c, HDL, LDL, TG, ACR, eGFR, glucose, insulin, QUICKI, HOMA-IR, HOMA-S, HOMA-B, and eGDR. For all combinations of parameters, the maximum values of the quality indicator of the classification model used exceed 80 [%], while the minimum values are below ACC = 40 [%]. Of all the presented combinations of parameters, the most important were selected, for which the classification allowed for obtaining results above ACC = 82 [%]. Table 5 summarizes the obtained results with the highest accuracy values for different configuration of parameters.

The results allowed for constructing sets of specific panels of biochemical indicators, which are the most useful in differentiating the metabolic status of patients with diabetes before and after six months of metformin treatment. Among these sets, variables differentiating with the highest accuracy the metabolic state of patients before and, after six-month metformin treatment, were: (1) vaspin, HbA1c, HDL, LDL, TG, insulin, and HOMA-B (ACC = 88 [%]); (2) vaspin, irisin, QUICKI, and eGDR (ACC = 86 [%]); as well as, (3) vaspin, irisin, LDL, HOMA-S, ACR, and eGFR (ACC = 86 [%]). Interestingly, the configuration with the minimal number of parameters and the highest possible differentiating power was based on vaspin and TG (ACC = 85 [%]) as well as vaspin and irisin (ACC = 82 [%]) results.

## 4. Discussion

The role of the adipokines/regulatory molecules in the modulation of metabolic processes related to obesity and the interrelations between the dysregulated expression of these substances in obesity states and the development of metabolic syndrome fully justify attempts to include their quantitative determination in the blood for the diagnosis of obesity-related diseases or assess the effects of the implemented therapy. However, previous studies provide conflicting information on the concentrations of adropin, irisin, and vaspin in the blood of T2DM patients [35,36,37,38,39].

### 4.1. Adropin Secretory Profile in Obese Controls and T2DM—The Influence of Six-Month Metformin Therapy, Sex Differences, Correlation With Biochemical Variables, Mechanism of Action

The results obtained in this study did not show significant differences in the plasma concentration of **adropin** in diabetic patients after six months of conventional metformin therapy in relation to the situation before treatment, and to the obese controls. Contradictory results of studies analyzing the plasma level of adropin in the course of T2DM have been published so far. Most of them indicate an increase in the circulating level of this peptide [38,39]. Hu and Chen [35] and Chen et al. [36] showed significantly lower levels of adropin in T2DM patients when compared to healthy subjects, additionally suggesting its possible participation in the pathogenesis and development of diabetic nephropathy. Significantly lower levels of circulating adropin in patients with type 2 diabetes as compared to healthy subjects were also confirmed in studies by Wu et al. [37]. They found that the low circulating level of this regulatory peptide turned out to be an independent prognostic factor for coronary atherosclerosis in both T2DM and healthy subjects. On the other hand, the results of Hosseini [38] and Ugur [39] contrast with the results of the study cited above. These authors have independently demonstrated that patients with T2DM were characterized by significantly higher plasma levels of adropin when compared to healthy individuals. This could be explained by a compensatory mechanism, a specific “feedback” reaction of adropin towards persistent hyperglycemia. It is believed that adropin can play a significant role in maintaining metabolic homeostasis, increasing the glucose utilization at the expense of fatty acids, improving glucose tolerance, and reducing insulin resistance [40,41,42]. Therefore, it seems that the relationship between plasma adropin, obesity rates, and circulating markers of lipid metabolism is more complex than previously thought. Adropin is a secreted protein abundantly expressed in brain as well as liver. A recent report revealed that adropin is also present in human plasma; however, the source and the mechanism of secretion in the circulation are elusive. It has been found that the expression of the adropin transcript in liver of mice is regulated by fasting and dietary macronutrients. Studies by Kumar et al. [3] revealed that hepatic expression of the Enho transcript is altered with obesity. Thus, serum adropin is also regulated by many factors i.e., fasting, dietary macronutrients, and obesity.

Similarly, the effect of gender on circulating adropin has not been clearly established. The results obtained in this study indicated a significantly higher adropin plasma level in obese healthy males as compared to obese healthy females. However, these changes have not been observed in males and females with T2DM before treatment and after six months of metformin. However gender factor was not variable influencing changes of adropin plasma level in obese diabetic patients. Our results obtained for obese non-diabetic subjects are consistent with the results that were obtained by Butler et al. [10] and Ghoshal et al. [42], in which obese males had higher plasma adropin concentration in relation to obese females. In diabetic patients, adropin was significantly increased in female when compared to males Korean T2DM patients [43]. Undoubtedly, the gender-specific regulation of adropin still needs to be explored in more detail. There is growing evidence demonstrating that circulating adropin levels depend upon diet preferences. It was shown that, in females but not in males, plasma adropin concentration positively correlated with fat intake. With an increase in the adropin level, a corresponding decrease in fat accumulation, plasma TG, and inflammation status were observed. Therefore, the proposed adropin may be used as a biomarker for predicting the risk of obesity and inflammation in T2DM [43].

We have also shown significant positive correlations between adropin and triglyceride and triglyceride/HDL levels in the group of patients with newly diagnosed type 2 diabetes. Butler et al. also presented evidence indicating the relationship between adropin and obesity [10]. They found that reduced adropin levels accompanied obesity and insulin resistance, while weight loss increased adropin levels. Experimental studies conducted by Gao et al. [32] confirmed that adropin treatment can enhance glucose tolerance and ameliorate insulin resistance in C57BL/6 mice with diet-induced obesity [32]. In muscle, adropin increased insulin-induced Akt phosphorylation and expression of GLUT4 on the cell surface and improved mitochondrial function in lipid metabolism by reducing incomplete fatty acid oxidation and increasing CoA/acetylCoA ratio. The mechanisms underlying these beneficial effects of adropin administration include suppression of an enzyme that is important in the metabolism of fatty acids—carnitine palmitoyltransferase-1B (CPT-1B) and a protein that facilitates transmembrane transport of free fatty acids—fatty acid translocase (FAT/CD36). Adropin administration also activates pyruvate dehydrogenase (PDH), an enzyme linking the glycolysis pathway with the citric acid cycle, and it reduces the activity of pyruvate dehydrogenase kinase-4 (PDH-4), which inhibits PDH. Alongside with these changes, adropin therapy down-regulated the gamma-1α receptor activated by the peroxisome proliferator, which regulates expression of Cpt-1b, Cd36 and Pdk-4 [32]. Therefore, adropin treatment appears to promote the preferential use of carbohydrates as an energy substrate, while reducing fatty acid oxidation; however, further research is still needed to finally clarify the molecular mechanisms that regulate the effects of adropin on carbohydrate-lipid metabolism in obesity.

### 4.2. Irisin Secretory Profile in Obese Controls and T2DM—Mode of Action, the Influence of Six-Month Metformin Therapy, Factors Affecting Plasma Irisin Level (BMI, Sex Differences), Correlation with Biochemical Variables

Another regulatory protein involved in ensuring the metabolic and energetic balance of the body, evaluated in this paper, was **irisin.** This novel hormone-like polypeptide is produced as a result of proteolytic cleavage of fibronectin type III domain-containing protein 5 (FNDC5) present in the membrane of myocytes as well as subcutaneous and visceral fat cells [14,44]. Irisin has been the subject of extensive research, which enabled to gain insight into its pleiotropic properties. Its association with obesity, insulin resistance, T2DM, metabolic syndrome, and dyslipidemia has been the subject of intensive research in recent years [45]. Strong limitation to the understanding of irisin mechanisms of action was the lack of knowledge about its receptor, which remained undiscovered for a long time. In 2018 Kim et al. identified the αV class of integrins, which are irisin receptors in osteocytes and adipose tissues [46]. Irisin plays a role of a thermogenic agent, promotes white-to-brown fat transdifferentiation, and also serves anti-obesity and anti-diabetic functions. That molecule facilitates glucose uptake by skeletal muscles, improves hepatic glucose and lipid metabolism, influencing a positive effect on hyperglycemia and hyperlipidemia and thereby acting as an insulin sensitizing hormone [33,34]. It was also reported that irisin promotes β cell proliferation through the ERK and p38 MAPK signaling pathways inhibits the high-glucose-induced apoptosis by regulating the expression of caspases, Bad, Bax, Bcl-2, and Bcl-xl, and improves pancreatic β cell function [47]. The protective action of irisin on β-cell lipid metabolism and inflammation under type 2 diabetic condition was confirmed in Zhang et al. studies [48]. Moreover, irisin also improved insulin secretion, inhibited apoptosis, and restored β-cell function-related gene expression in isolated mouse islets under glucolipotoxic conditions [48]. The beneficial role of irisin in glucose metabolism in T2DM has also been associated with the regulation of p38 MAPK signaling by β-arestin type 2 [49]. Mitogen-activated serine-threonine protein kinases (MAPKs) are involved in the transmission of signals that regulate gene expression, proliferation, and apoptosis. Irisin and the cytosolic protein, type 2 β-arestin, activate p38 MAPK, which phosphorylates membrane and cytoplasmic proteins and, after translocation to the cell nucleus, also various transcription factors. The overexpression of β-arestin 2 increases irisin-stimulated cellular glucose uptake by enhancing the function of GLUT-4 transporter proteins [49]. Under normal metabolic conditions, muscles are acknowledged as the main source of circulating irisin [50]. Circulating irisin was found to be positively associated with parameters of adiposity, such as BMI, with fat mass being suggested as a potential determinant of circulating irisin in humans. In mice, it has been calculated that approximately 28% of circulating irisin originates from adipose tissue. However, the relative contribution of muscle or adipose tissue to irisin secretion varies depending on adipose tissue dysfunction associated with VAT accumulation. Roca Rivada et al. hypothesized that pathological conditions, like obesity, make adipose tissue more relevant for FNDC5/ irisin secretion than other body tissues [51]. Most studies showed a decrease in irisin plasma levels in T2DM patients compared to healthy people [7,52,53]. Choi et al. [53] found that reduced plasma levels of irisin in patients with newly diagnosed type 2 diabetes correlated with BMI, HbA1c, and triglyceride levels, showing no correlation with WHR, fasting glucose and insulin, lipid profile, or HOMA-IR. Additionally, the Moreno-Navarrete et al. [54] study showed that plasma levels of irisin and the expression of the FNDC5 gene in muscle and fat tissue in obese patients with type 2 diabetes and obesity are reduced. Very similar results were obtained by Shelbaya et al. [55]. In addition to the lower concentration of irisin in the blood of patients with type 2 diabetes compared to healthy people, they revealed the negative correlations with creatinine, systolic, and diastolic blood pressure, BMI, ACR, and HbA1c [55]. Bonfante et al. [56] found in their research that a higher level of irisin is associated with a better metabolic profile of obese people.

Our results showed that in obese patients with T2DM, both before and after providing metformin treatment, irisin was present in higher concentrations compared to the results of obese people. On the other hand, six-month metformin therapy resulted in an increase in the concentration of the evaluated regulatory molecule in relation to the pre-treatment situation. Consistent with our results are studies by Rana et al. [45], which showed elevated plasma levels of irisin in T2DM in obese patients when compared to healthy people. Also, Al-Daghri et al. [57] and García-Fontana et al. [58] showed significantly higher levels of irisin in the blood of patients with type 2 diabetes. The latter explain this fact by a compensatory mechanism related to the modulation of the metabolic state of obese T2DM patients [58]. Altogether, that evidence, in line with the increased concentration of irisin in T2DM patients before and after treatment found in this study, may be an adaptive reaction in relation to the decreasing insulin sensitivity in patients in the initial stage of the disease [58]. It has been also suggested that increased circulating irisin in obesity is a compensatory response to obesity-induced disturbed metabolism, such as decreased insulin level [59]. Alternatively, ”irisin resistance” may be another description for increased levels of irisin in obesity [60]. These findings point toward a compensatory mechanism in the early diabetes and possibly ‘irisin-resistant’ state, where increased amounts of irisin are secreted in an attempt to increase energy expenditure by browning of WAT or other yet unidentified effects in skeletal muscle [61]. On the other hand increased circulating irisin in T2DM patients after six-month treatment may be associated with the metabolically beneficial “protective” effect of the metformin therapy used. Studies have shown that conventional metformin therapy enhances the expression of irisin in skeletal muscle cells and contributes to the increase of its plasma concentration independent of the activation of the AMPK pathway [26]. Yang et al. [27] showed that PGC-1α is the regulator of these processes. Although stimulation of the synthesis of irisin as a new molecular mechanism of metformin action requires further research and verification, it will probably remain a matter of the near future.

The association of circulating irisin with metabolic risk factors in patients withT2DM has also been the subject of numerous studies. Previous studies have shown that plasma irisin concentrations were correlated with anthropometric and metabolic markers of obesity and type 2 diabetes; however, conflicting results were obtained [7,62,63]. Some reports suggest a positive correlation between irisin and BMI [64], whilst other studies have found either no association [65,66] or a negative association [67]. In this study, no correlation was found between circulating irisin and BMI both in obese and nondiabetic patients as well as in obese patients with type 2 diabetes before and after six months of metformin therapy. On the other hand, the results found in our study are consistent with the results of most studies regarding the relationship of circulating irisin with both insulin sensitivity indices and carbohydrate metabolism parameters. It is worth noting that the strongest correlations of circulating irisin found in this study were related to HbA1c in the obese T2DM group and to the QUICKI index in the T2DM group after six months of metformin therapy. Therefore, it appears that plasma profile of irisin may be related with the level of glycemic control and insulin resistance; however, additional large scale studies are needed in order to elucidate this phenomenon. The effect of gender on the plasma profile of irisin is also not fully established. Lower circulating irisin was reported in obese males than in obese females [68,69], which could result from the variations in hormonal levels as well as from the differences in the distribution of fat in males and females. In this study no gender-dependent differences for irisin plasma level were found. Our results are, in part, consistent with the results that were obtained by Jameel et al. who did not show significant differences in circulating irisin levels between healthy men and healthy women with a body mass index (BMI) in the healthy range [66].

### 4.3. Vaspin Secretory Profile in Obese Controls and T2DM—Gender-Related Differences, the Influence of 6-Month Metformin Therapy, Correlation With Biochemical Variables, Mechanism of Action

**Vaspin**, a visceral adipose tissue-derived serine protease inhibitor with insulin-sensitizing effects, which belongs to the serpin superfamily, was another regulatory molecule analyzed in this study that plays an important role in maintaining metabolic homeostasis in obesity state. This peptide hormone synthesized mainly in visceral and subcutaneous adipose tissue has been the subject of intensive research in recent years. Studies indicate the important role of vaspin in the pathogenesis of metabolic disorders that are associated with obesity in the course of type 2 diabetes. It has been shown so far that the plasma concentration of vaspin in the course of T2DM may be higher [21,70,71,72,73,74,75], lower [23,76,77], or similar [18,78,79] to the plasma concentrations of this peptide in subjects with normal glucose tolerance. The reason for above discrepancies may be that the studies are related to type 2 diabetes at various stages. Adipose tissue during long-lasting T2DM, often with the presence of long-term complications, has a different secretory profile when compared to adipose tissue of patients with newly diagnosed T2DM.

The results that were obtained in this study indicated a significantly lower circulating vaspin concentration in obese patients with newly diagnosed T2DM before the implementation of metformin therapy, as compared to obese controls. On the other hand, six-month metformin therapy resulted in a 48 [%] increase in this serpine plasma level compared to the pre-treatment situation. Our results are consistent with the results obtained by Yan et al. [76] and Castro et al. [77], in which T2DM patients had lower plasma vaspin concentration in relation to healthy individuals. Many factors can affect the plasma profile of that serpine.

So far, it has been shown that circulating vaspin level appears to be dependent on insulin sensitivity and glucose control. Some studies indicated a sexual dimorphism in circulating vaspin [18,80,81], while others do not confirm gender-related differences [82,83] Youn et al. [18] found that the level of plasma vaspin in females was significantly elevated compared to males in subjects with normal glucose tolerance, but this difference was not observed in diabetic patients [18]. Our research indicated that the plasma vaspin concentrations were not significantly different between obese males and females patients with T2DM and control group. Contradictory results concerning gender-related changes in circulating vaspin may be due to different patient populations in these studies or other factors affecting vaspin expression, such as diet or physical exercise [84]. A genome-wide association study conducted by Breitfeld at al. identified several single nucleotide polymorphisms (SNPs) in the vaspin locus of 14th chromosome associated with plasma vaspin levels. Thus, genetic variations are another most likely reason for the variability of serum vaspin [85].

Most studies in humans have shown a positive correlation between vaspin gene expression and serum levels, and metabolic syndrome parameters. The confirmation of the above thesis were the results of Pala et al. [86], indicating that increased levels of plasma vaspin were associated with a reduced risk of insulin resistance, regardless of BMI, as well as the results of studies conducted by Hao et al. [73] and Jian et al. [23], indicating a relationship between plasma vaspin concentration and BMI, glycemia, fasting insulin, and HOMA-IR. In turn, Jian et al. [23] demonstrated that low plasma vaspin concentration is a risk factor for T2DM progression and it is associated with a faster need for implementation of insulin therapy.

We found statistically significant positive correlations between vaspin plasma concentration and HOMA-IR, fasting insulin and HOMA-B, as well as a negative correlation between plasma vaspin level and HOMA-S in untreated obese patients with type 2 diabetes. After a six-month treatment with metformin, a negative relationship was found between plasma vaspin concentration and QUICKI index. In the light of the results obtained, it is worth emphasizing that the administration of recombinant vaspin to mice with diet-induced obesity led to an improvement in glucose tolerance and an increase in insulin sensitivity [87,88,89]. A confirmation of the above thesis is the results of additional statistical analyzes between the changes in plasma adipocytokines during metformin treatment and changes in values of insulin resistance indicators following six-month metformin therapy. Our results showed that metformin therapy was related with parallel decreases in HOMA-IR and HOMA-B. The obtained results may provide clinically useful information in assessing metabolic changes, associated with the metformin therapy.

### 4.4. Vaspin—Mode of Action

The relationship between circulating vaspin levels and insulin sensitivity parameters indicates the important role of this molecule in the pathogenesis of metabolic disorders associated with obesity, although mechanism of vaspin action remains under investigation. Human kallikrein 7 (hK7) was the first protease known as “target” of this bioactive molecule, inhibited by classical serpin mechanism. HK7 is known to be engaged in the process of insulin degradation exhibiting the ability to cleave human insulin within A- and B-chain [90]. Research that was carried out by Zieger at al. [90] on the experimental model of obesity provided the first evidence of a significant contribution of KLK7 to glucose metabolism. It has been established that the inhibition of hK7 in adipose tissue improves insulin sensitivity, increases energy expenditure, and reduces weight gain. Moreover, it enhances the activation of macrophages and induces secretion of pro-inflammatory cytokines. Thus, by inhibiting kallikrein 7, vaspin reduces local, chronic inflammation, and pathological expansion of adipose tissue, partially alleviating the adverse effects of obesity, which are caused by the use of high-fat diet [90]. It was also found that vaspin enhances the expression of the type 2 insulin receptor substrate (IRS-2) mRNA and increases the level of IRS-2 total protein, a critical element in insulin-signaling pathways. [19]

In addition, this regulatory peptide is associated with the activation of AMP, Akt, mTOR (mammalian target of rapamycin kinase), and p70S6K (ribosomal protein S6 kinase beta-1) kinases. AMPK is involved in the regulation of carbohydrate-lipid metabolism, being one of the main factors responsible for maintaining energy homeostasis. Akt is the main signal transmitter in the phosphatidylinositol 3-kinase pathway (PI3K) and it plays an important role in regulating insulin signaling pathway. In turn, mTOR and p70S6K are enzymes that integrate many cell signaling pathways, including the insulin one, the dysregulation of which may be involved in the pathogenesis of insulin resistance and type 2 diabetes. Prolonged activation of mTOR can activate the p70S6K leading to increased phosphorylation of IRS and down regulation of PI3K/Akt, which is involved in insulin resistance. The study conducted by Liu et al. confirmed that vaspin can relieve insulin resistance of islet β cells through the insulin signaling pathway of IRS-2/PI3K/Akt/mTOR/p70S6K. [19]. This research has also shown that in addition to insulin sensitizing properties, vaspin exhibits anti-inflammatory action. It can suppress proinflammatory cytokine mediated activation of NF-κB. Inflammatory factors can interfere with insulin signaling pathway of IRS/PI3K/Akt and, thus, promote insulin resistance. Nakatsuka et al. [17] have demonstrated that vaspin exerts its anti-inflammatory action through binding to 78-kDa glucose-regulated protein (GPR78), a glucose-regulated protein, which, under the influence of stress of the endoplasmic reticulum, moves to the cell membrane. Serving as a ligand for a cell-surface GRP78 anion channel complex in endothelial cells, vaspin exerts antiapoptotic, proliferative, and protective effects on vascular walls in experimentally induced diabetes mellitus [17]. Moreover, a recent study has reported that vaspin treatment reduces the NF-κB mRNA and protein levels [17]. Liu et al. [19] study also confirmed that vaspin inhibits the inflammation of pancreatic β cells through NF-κB signaling pathway, protecting β cells from damage and improving β cell function. The inhibitory effect of this visceral adipose tissue-derived serpin on the expression of NF-κB involved in many cellular processes appears to be a potential mechanism underlying the regulatory action of vaspin in obese patients in the course of T2DM [19,91].

In addition, research indicates that vaspin increases the bioavailability of nitric oxide by reducing dimethylarginine in endothelial cells [92,93], which seems to be important in the aspect of preventing the microvascular complications of type 2 diabetes. Undoubtedly, vaspin plays an important role in regulating glucose metabolism. Therefore, understanding mechanism of its action might support the development of novel etiology-based treatment strategies, targeting metabolic and glucose tolerance disorders.

### 4.5. Three-Parameter Assessment and Visualization of the Relationship Between Biochemical Variables—Diagnostic Panels Useful in Early Detection of T2DM and Differentiating the Obesity Phenotype with Normal Metabolic Profile from T2DM Obese Patients

The conducted discriminant analysis using bioinformatic tools indicated that most important biochemical parameters in constructed tree-parameter diagnostic panels were (1) plasma adropin, HOMA-B, HOMA-S, (2) plasma adropin, irisin, and vaspin, and (3) plasma irisin, QUICKI, and HOMA-IR. Significant changes in the metabolism of the studied adipocytokines were found in obese patients with type 2 diabetes, which may be particularly useful at an early stage of carbohydrate and fat metabolism abnormalities, leading to the development of diabetes.

Moreover, with the use of discriminant analysis, we selected the most important diagnostic parameters, differentiating the metabolic state of T2DM patients before the implementation of metformin therapy, and six months after its application. So far, this has not been analyzed in the population of T2DM patients. Plasma irisin and vaspin quantification in obese patients with type 2 diabetes, effectively showed that a six-month metformin therapy significantly influences plasma concentration of analyzed regulatory molecules, suggesting their potential diagnostic utility in monitoring metabolic changes associated with the introduction of pharmacological treatment in type 2 diabetic patients. On the base of three-parameter discriminant analysis, we have found that following panels of markers: (1) irisin, HbA1C, and eGDR; (2) adropin, irisin, and vaspin; and, (3) irisin, ACR, and eGFR best reflect the effect of the therapy on endocrine activity of adipose tissue.

Therefore, the conducted studies indicated that, despite the fact that single determinations of the concentration of individual adipocytokines during metformin therapy may not show up a parallel changes with the% changes of biochemical and metabolic status parameters, the three-parameter panels used provide clinically useful information in assessing metabolic changes, associated with the metformin therapy.

### 4.6. Multifactorial Discriminant Analysis—Diagnostic Panels Based on Irisin and Vaspin Plasma Levels Contribute Clinically Relevant Information Concerning the Effectiveness of Metformin Treatment in T2D Patients

The use of multifactorial discriminant analysis based on HbA1c, HDL, LDL, TG, ACR, eGFR, glucose, insulin, QUICKI, HOMA-IR,% S,% B, and eGDR allowed to put forward specific panels of biochemical indicators differentiating patients from T2DM before and after metformin therapy. The highest accuracy in differentiating patients’ metabolic status was found in the following sets: (1) vaspin, HbA1c, HDL, LDL, TG, insulin, and HOMA-B; (2) vaspin, irisin, QUICKI and eGDR; and, (3) vaspin, irisin, LDL, HOMA-S, ACR, and eGFR. However, due to the fact that a smaller number of variables in the diagnostic panel translates directly into a reduction in outpatient costs, we selected from the previously proposed variables, the panel with the minimum number of parameters with the highest possible differentiating power. Such configurations included a panel based on the plasma determination of vaspin and TG concentration (ACC = 85 [%]) and the concentration of irisin and vaspin (ACC = 82 [%]) in the blood plasma.

### 4.7. Limitations of the Study

This study has some limitations. Firstly, the number of obese diabetic subjects included into the study was relatively small, however it allowed for finding statistically significant differences between variables. Only 16 men took part in the study, which limited the analysis with respect to gender. Additional large scale studies in this domain and extended period of observation should be performed to confirm our observation. Secondly, circulating adipokine profile is associated with many factors, including genetic variants associated with the serum adipokine profile as well as physical activity, body composition, and diet. In our study, only insulin resistance and carbohydrate-lipid metabolism were examined. Genetic variants associated with the serum concentration of specific adipokines and hormonal peptides secreted from peripheral organs should be taken into account in future analyses because they can promote metabolic disturbances or, with regard to the “protective” allele, exert beneficial effects [4,85]. Lastly, we did not compare the results with normal-weight subjects with insulin resistance, but without diabetes, which could link the effect of insulin resistance alone on the plasma profile of analyzed adipokines. Recent studies suggest that proinflammatory factors produced by fat cells (cytokines, adipokines) and peripheral tissues impair glucose metabolism by inducing chronic inflammation and induce insulin resistance, irrespective of body weight and fat distribution. Thus, the knowledge of plasma profile of adipokines and bioactive molecules produced by peripheral tissues and involved in the carbohydrate-lipid metabolism could be especially important in estimating the risk of diabetes in subjects with normal body weight.

## 5. Conclusions

This study had significant power to detect significant association between circulating adipokines and various metabolic parameters and for the first time allowed proposing three- and multi-parameter diagnostic panels which can distinguish between obese subjects and obese patients with type 2 diabetes. These data could be helpful in the early diagnosis of T2DM and they indicate the possibility of the development of diabetes in obese individuals. Moreover, diagnostic panels based on circulating irisin and vaspin have been shown to be useful markers for both early detection of T2DM and monitoring the effects of the implemented treatment with metformin; however further large-scale studies are required in order to gain more insight into the diagnostic role of analyzed adipokines/myokines in T2DM.

## Figures and Tables

**Table 1:** Clinical characteristics of control subjects and obese patients with type 2 diabetes mellitus.

Parameters	Control Subjects	Obese Patients with Type 2 Diabetes Mellitus		[%] Change Following Therapy
		Before Metformin Therapy	After the 6-Month Metformin Treatment	
Female/Male (n)	20 (11F/9M)	40 (24F/16M)	40 (24F/16M)	
Age (yr)	56.05 ± 4.99	59.28 ± 7.29	59.88 ± 7.29	
BMI (kg/m^2^)	29.59 ± 6.22	32.76 ± 4.83 ^a^	32.13 ± 5.06 ^a^	−1.92
WHR (cm^2^)	0.95 ± 0.07	0.98 ± 0.06	0.97 ± 0.06	−1.02
Glucose (mg/dL)	90.50 ± 5.09	113.94 ± 12.06 ^a^	114.22 ± 14.38 ^a^	+0.25
HbA1c (%)	5.21 ± 0.39	6.26 ± 0.61 ^a^	6.33 ± 0.79 ^a^	+1.12
Triglycerides (mg/dL)	162.07 ± 50.01	176.84 ± 93.14	142.42 ± 66.25	−19.46
Cholesterol (mg/dL)	181.23 ± 27.27	196.66 ± 44.87	182.29 ± 29.10 ^d^	−7.31
HDL (mg/dL)	46.80 ± 8.53	50.10 ± 14.77	51.59 ± 14.55	+2.97
LDL (mg/dL)	115.78 ± 28.44	113.79 ± 35.54	99.21 ± 27.17 ^b,c^	−12.81
TG/HDL ratio	3.65 ± 1.47	4.16 ± 2.61	3.12 ± 1.98	−25.00
sCr (mg/dL)	0.85 ± 0.10	0.79 ± 0.13	0.85 ± 0.17 ^d^	+7.59
uCr (mg/dL)	110.60 ± 65.43	100.74 ± 56.90	105.51 ± 52.54	+4.73
uAlbumin (μg/mL)	4.66 ± 3.81	5.99 ± 5.32	6.05± 5.65	+1.00
ACR (μg/mg Cr)	5.63 ± 4.51	6.94 ± 5.39	6.02± 4.56	−13.26
eGFR (mL/min/1.73m^2^)	90.89 ± 13.74	90.37 ± 13.84	84.33± 16.69 ^d^	−6.68
Insulin (μIU/mL)	13.15 ± 9.72	10.53 ± 6.43	9.52 ± 5.12	−9.59
HOMA-IR2	1.16 ± 0.73	1.46 ± 0.83	1.29 ± 0.68 ^d^	−11.64
HOMA-B	135.59 ± 58.60	78.16 ± 37.88 ^a^	69.06 ± 32.86 ^a^	−11.64
HOMA-S	85.05 ± 61.96	93.50 ± 55.60	99.44 ± 54.01	+6.35
QUICKI	0.36 ± 0.06	0.35 ± 0.05	0.35 ± 0.04	0.00
eGDR	9.78 ± 0.93	7.46 ±1.77 ^a^	7.88 ± 1.98 ^a^	+5.63
Systolic pressure [mmHg]	122.50 ± 6.98	135.69 ± 10.71 ^a^	131.67 ± 10.95 ^a^	−2.96
Diastolic pressure [mmHg]	81.25 ±3.41	81.67 ± 5.07	78.06 ± 7.39 ^d^	−4.42

Values given as mean ± SD; F, female; M, male; BMI, body mass index; WHR, waist-hip ratio; HbA1c, glycated haemoglobin; HDL, high density lipoproteins; LDL, low density lipoproteins; sCr, creatinine in serum; uCr, creatinine in urine; uAlbumin, albumin in urine; ACR, albumin/creatinine ratio; eGFR, estimated glomerular filtration rate; HOMA-IR2, homeostatic model assessment of insulin resistance; HOMA-B, homeostatic model assessment of B cell function; HOMA-S, homeostatic model assessment of insulin sensitivity; QUICKI—quantitative insulin sensitivity check index; eGDR, estimated glucose disposal rate. ^a^ *p* < 0.0005, compared to controls; ^b^ *p* < 0.05, as compared to controls; ^c^ *p* < 0.001, compared to diabetic patients before metformin therapy; ^d^ *p* < 0.05, as compared to diabetic patients before metformin therapy.

**Table 2:** Concentrations of plasma adropin, irisin and vaspin in control subjects and obese patients with type 2 diabetes mellitus.

Parameters	Control Subjects	Obese Patients with Type 2 Diabetes Mellitus		[%] Change Following Therapy
		Before the Implementation of Metformin Therapy	After the 6-Month of Metformin Treatment	
**Adropin (ng/mL)**				
All	0.75 (0.62–1.06)	0.86 (0.61–1.07)	0.74 (0.52–0.93)	−13.95
F	0.56 (0.40–0.70)	0.88 (0.62–1.06)	0.74 (0.51–0.90)	−15.91
M	0.79 (0.71–1.13) ^e^	0.76 (0.52–1.08)	0.74 (0.52–0.97)	−2.63
**Irisin (μg/mL)**				
All	4.99 (3.29–6.16)	8.83 (5.63–12.39) ^a^	15.28 (11.39–19.78) ^a,d^	+73.05
F	4.99 (3.45–6.28)	8.78 (5.81–13.91)	18.10 (12.20–20.14)	+106.15
M	4.73 (3.28–5.91)	9.57 (5.04–11.06)	13.86 (10.02–15.80)	+44.83
**Vaspin (ng/mL)**				
All	0.35 (0.21–0.99)	0.12 (0.07–0.29) ^b^	0.29 (0.17–0.66) ^c^	+141.67
F	0.38 (0.21–0.53)	0.11 (0.07–0.21)	0.29 (0.20–0.45)	+163.64
M	0.31 (0.11–0.82)	0.15 (0.07–0.38)	0.31 (0.13–0.70)	+106.67

Values given as median and interquartile (25th–75th percentile) range. ^a^ *p* <0.0005, compared to controls; ^b^ *p* < 0.005, compared to controls; ^c^ *p* < 0.000001, compared to diabetic patients before metformin therapy; ^d^ *p* < 0.000005, compared to diabetic patients before metformin therapy; ^e^ *p* < 0.005, compared to females from control group.

**Table 3:** Correlation between plasma adipocytokines and anthropometric parameters, renal function indices, insulin resistance indicators and carbohydrate-lipid metabolism parameters in obese patients with type 2 diabetes before implementation of metformin therapy and after a 6-month therapy.

Parameters	Obese T2DM Patients Before Metformin Therapy		
	Adropin (ng/mL)	Irisin (μg/mL)	Vaspin (ng/mL)
Weight (kg)	−0.086 ^NS^	−0.217 ^NS^	0.151 ^NS^
BMI (kg/m^2^)	0.127 ^NS^	−0.111 ^NS^	−0.044 ^NS^
ACR (mg/mg Cr)	−0.014 ^NS^	−0.264 ^NS^	−0.209 ^NS^
eGFR (mL/min/1.73m^2^)	0.1 ^NS^	−0.304 (*p* = 0.045)	−0.251 ^NS^
Insulin (mIU/mL)	0.1 ^NS^	0.222 ^NS^	0.491(*p* = 0.007)
HOMA-IR2	0.164 ^NS^	0.177 ^NS^	0.437 (*p* = 0.02)
HOMA-S	−0.152 ^NS^	−0.160 ^NS^	−0.445 (*p* = 0.017)
HOMA-B	0.167 ^NS^	0.089 ^NS^	0.352 (*p* = 0.006)
QUICKI	−0.144 ^NS^	−0.104 ^NS^	−0.292 ^NS^
eGDR	−0.124 ^NS^	0.073 ^NS^	−0.084 ^NS^
Glucose (mg/dL)	−0.018 ^NS^	−0.008 ^NS^	−0.029 ^NS^
HbA1c (%)	0.255 ^NS^	−0.430 (*p* = 0.008)	0.172 ^NS^
Cholesterol (mg/dL)	0.124 ^NS^	−0.014 ^NS^	0.177 ^NS^
HDL (mg/dL)	−0.161 ^NS^	0.137 ^NS^	−0.081 ^NS^
LDL (mg/dL)	−0.132 ^NS^	−0.042 ^NS^	0.135 ^NS^
Triglycerides (mg/dL)	0.479 (*p* = 0.003)	−0.193 ^NS^	0.278 ^NS^
TG/HDL	0.382 (*p* = 0.021)	−0.203 ^NS^	0.213 ^NS^
**Parameters**	**Obese T2DM Patients After the 6-Month Metformin Therapy**		
	**Adropin (ng/mL)**	**Irisin (** **μg/mL)**	**Vaspin (ng/mL)**
Weight (kg)	0.143 ^NS^	−0.253 ^NS^	0.435 (*p* = 0.008)
BMI (kg/m^2^)	−0,087 ^NS^	−0,045 ^NS^	0,244 ^NS^
ACR (mg/mg Cr)	−0.222 ^NS^	0.131 ^NS^	−0.0154 ^NS^
eGFR (mL/min/1.73m^2^)	−0.261 ^NS^	−0.072 ^NS^	−0.219 ^NS^
Insulin (mIU/mL)	0.102 ^NS^	−0.115 ^NS^	0.347 ^NS^
HOMA-IR2	0.108 ^NS^	−0.087 ^NS^	0.349 ^NS^
HOMA-S	−0.097 ^NS^	0.082 ^NS^	−0.345 ^NS^
HOMA-B	−0.004 ^NS^	−0.249 ^NS^	0.305 ^NS^
QUICKI	−0.054 ^NS^	0.349 (*p* = 0.039)	−0.372 (*p* = 0.025)
eGDR	−0.015 ^NS^	0.125 ^NS^	−0.04 ^NS^
Glucose (mg/dL)	0.193 ^NS^	0.241 ^NS^	−0.059 ^NS^
HbA1c (%)	−0.232 ^NS^	−0.195 ^NS^	0.055 ^NS^
Cholesterol (mg/dL)	−0.015 ^NS^	−0.006 ^NS^	−0.091 ^NS^
HDL (mg/dL)	0.03 ^NS^	−0.004 ^NS^	−0.021 ^NS^
LDL (mg/dL)	−0.213 ^NS^	−0.124 ^NS^	−0.081 ^NS^
Triglycerides (mg/dL)	0.025 ^NS^	−0.033 ^NS^	0.188 ^NS^
TG/HDL	0.026 ^NS^	0.018 ^NS^	0.092 ^NS^

Spearman rank correlation coefficients; NS—not significant.

**Table 4:** Accuracy for three-parameter discriminant analysis which allows to differentiate the patients with metabolically compensated obesity and Type 2 diabetes (T2DM) obese patients (Part A), as well as T2DM obese patients before and after 6-month metformin therapy (Part B), based on adropin, irisin or vaspin values and selected variables.

Part A		Part B	
Three-Parameter Sets of Variables Which Allow to Differentiate the Healthy Subjects and T2DM Patients	ACC [%]	Three-Parameter Sets of Variables Which Allow to Differentiate the T2DM Patients before and after 6-Month Metformin Therapy	ACC [%]
adropin, irisin and vaspin	94	adropin, irisin and vaspin	69
adropin, QUICKI and HOMA-IR	71	adropin, QUICKI and HOMA-IR	48
irisin, QUICKI and HOMA-IR	90	irisin, QUICKI and HOMA-IR	57
vaspin, QUICKI and HOMA-IR	63	vaspin, QUICKI and HOMA-IR	59
adropin, ACR and eGFR	80	adropin, ACR and eGFR	44
irisin, ACR and eGFR	75	irisin, ACR and eGFR	68
vaspin, ACR and eGFR	73	vaspin, ACR and eGFR	50
adropin, HbA1c and eGDR	86	adropin, HbA1c and eGDR	57
irisin, HbA1c and eGDR	88	irisin, HbA1c and eGDR	80
vaspin, HbA1c and eGDR	82	vaspin, HbA1c and eGDR	56
adropin, HOMA-B and HOMA-S	95	adropin, HOMA-B and HOMA-S	27
irisin, HOMA-B and HOMA-S	75	irisin, HOMA-B and HOMA-S	50
vaspin, HOMA-B and HOMA-S	67	vaspin, HOMA-B and HOMA-S	50
adropin, insulin and TG/HDL	77	adropin, insulin and TG/HDL	59
irisin, insulin and TG/HDL	66	irisin, insulin and TG/HDL	45
vaspin, insulin and TG/HDL	80	vaspin, insulin and TG/HDL	63

ACC—accuracy.

**Table 5:** Multiparameter discriminant analysis which allow to differentiate the metabolic state of T2DM obese patients before and after metformin treatment.

Variables in the Diagnostic Panel													ACC [%]
Vaspin	-	HbA1c	HDL	LDL	TG	Insulin	-	HOMA-B	-	-	-	-	88
Vaspin	Irisin	-	-	-	-	-	QUICKI	-	-	-	-	eGDR	86
Vaspin	Irisin	-	-	LDL	-	-	-	-	HOMA-S	ACR	eGFR	-	86
Vaspin	Irisin	-	-	-	-	-	-	-	-	-	-	-	82
Vaspin	Irisin	HbA1c	HDL	LDL	TG	-	QUICKI	-	-	-	-	-	85
Vaspin	-	-	-		TG	-	-	-	-	-	-	-	85
Vaspin	-	-	HDL	-	TG	-	QUICKI	-	-	-	-	-	85
Vaspin	-	-	HDL	LDL	-	-	QUICKI	-	-	-	-	eGDR	85
Vaspin	-	HbA1c	HDL	LDL	TG	-	QUICKI	-	-	-	-	-	85
Vaspin	-	HbA1c	HDL	LDL	TG	Insulin	QUICKI	HOMA-B	-	-	-	-	85
Vaspin	-	HbA1c	HDL	-	TG	-	QUICKI	-	-	-	-	-	85

In red: the configurations with the minimum number of parameters and the highest possible differentiating power.
